# Effects of habitat management on rodent diversity, abundance, and virus infection dynamics

**DOI:** 10.1002/ece3.10039

**Published:** 2023-04-25

**Authors:** Nathaniel Mull, Amy Schexnayder, Abigail Stolt, Tarja Sironen, Kristian M. Forbes

**Affiliations:** ^1^ Department of Biological Sciences University of Arkansas Fayetteville Arkansas USA; ^2^ Department of Virology University of Helsinki Helsinki Finland; ^3^ Department of Veterinary Biosciences University of Helsinki Helsinki Finland

**Keywords:** grassland, land management, orthohantavirus, prairie, prescribed burn, restoration

## Abstract

As anthropogenic factors continue to degrade natural areas, habitat management is needed to restore and maintain biodiversity. However, the impacts of different habitat management regimes on ecosystems have largely focused on vegetation analyses, with limited evaluation of downstream effects on wildlife. We compared the effects of grassland management regimes (prescribed burning, cutting/haying, or no active management) on rodent communities and the viruses they hosted. Rodents were trapped in 13 existing grassland sites in Northwest Arkansas, USA during 2020 and 2021. Rodent blood samples were screened for antibodies against three common rodent‐borne virus groups: orthohantaviruses, arenaviruses, and orthopoxviruses. We captured 616 rodents across 5953 trap nights. Burned and unmanaged sites had similarly high abundance and diversity, but burned sites had a higher proportion of grassland species than unmanaged sites; cut sites had the highest proportion of grassland species but the lowest rodent abundance and diversity. A total of 38 rodents were seropositive for one of the three virus groups (34 orthohantavirus, three arenavirus, and one orthopoxvirus). Thirty‐six seropositive individuals were found in burned sites, and two orthohantavirus‐seropositive individuals were found in cut sites. Cotton rats and prairie voles, two grassland species, accounted for 97% of the rodents seropositive for orthohantavirus. Our study indicates that prescribed burns lead to a diverse and abundant community of grassland rodent species compared with other management regimes; as keystone taxa, these results also have important implications for many other species in food webs. Higher prevalence of antibodies against rodent‐borne viruses in burned prairies shows an unexpected consequence likely resulting from robust host population densities supported by the increased habitat quality of these sites. Ultimately, these results provide empirical evidence that can inform grassland restoration and ongoing management strategies.

## Graphical abstract

Burned and unmanaged sites had similarly high rodent abundance and diversity, but burned sites had a greater proportion of grassland species than unmanaged sites; cut sites had the highest proportion of grassland species but the lowest rodent abundance and diversity. Thirty‐six individuals seropositive for an orthohantavirus, arenavirus, or orthopoxvirus were found in burned sites, and two orthohantavirus‐seropositive individuals were found in cut sites.

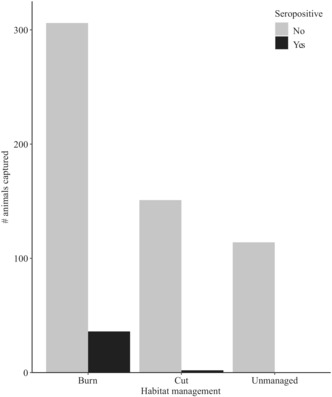

## 1 INTRODUCTION

Healthy ecosystem functioning is usually dependent on biodiversity, including species, genetic, and even parasite diversity (Cardinale et al., 2006; Duffy, 2009; Hughes et al., 2008; Winder & Shamoun, 2006). However, biodiversity continues to be negatively impacted by a variety of phenomena, including climate change, pollution, and most considerably, changes in land cover and land use (Haines‐Young, 2009; Sala et al., 2000; Young et al., 2010). This is exemplified by grasslands in the United States, where approximately 70% of total historical prairie habitat and 90% of tallgrass prairie habitat has been lost (Samson et al., 2004). Habitat management is a key component of efforts to restore and maintain grassland biodiversity, but outcomes vary depending on the management strategies employed (Haddock et al., 2007; Haines‐Young, 2009; Turner II et al., 2007).

In light of the need for large‐scale restoration and protection in grassland habitats (Gerla et al., 2012), research on the effects of different management regimes on grassland vegetation is accumulating (e.g., Feher et al., 2021; Newbold et al., 2020). Quantifying changes in vegetation provides valuable insight into the benefits of different management regimes on habitat quality. However, there has been little research on the downstream effects of grassland management practices on animal communities, and most available studies have focused on livestock (Paudel et al., 2021) or the integration of wildlife habitat into agricultural systems (e.g., Burkhalter, 2013; Lukens et al., 2020). Given that a key objective of management is to restore and enhance species diversity (Newbold et al., 2020), studies are needed to identify the broader effects of different management regimes on wildlife diversity.

Species‐rich taxa such as rodents are highly effective systems to measure diversity and infer ecosystem health (Avenant, 2011; Fernández et al., 2021; Loggins et al., 2019). Rodents are the most diverse mammalian taxon, comprising approximately 40% of mammal species worldwide (Burgin et al., 2018), and play crucial roles in ecosystems, including both bottom‐up (e.g., seed dispersal; Sunyer et al., 2013) and top‐down (i.e., common prey; Geng et al., 2009) processes. Because many rodent species have a fast pace of life strategy (i.e., r‐selected), their communities also quickly respond to changes in the environment (Zúñiga et al., 2020). For example, female hispid cotton rats (*Sigmodon hispidus*), a common grassland rodent in the USA, produce an average of 5.6 Litters/year and up to 12 pups/litter (Clark, 1972; Espinoza & Rowe, 1979).

Despite rodents being an integral part of ecosystems, they also carry many pathogens that can spillover and cause disease in humans (zoonoses; Begon, 2003; Dahmana et al., 2020; Meerburg et al., 2009). Thus, understanding the impacts of habitat management on pathogens is relevant to both wildlife and human health. Infection dynamics are often shaped by the characteristics of individual hosts and their populations. Notably, many pathogens require a minimum host abundance or density to persist in populations (density threshold; Lloyd‐Smith et al., 2005), and transmission rates often increase as abundance rises (density‐dependent transmission; Anderson & May, 1978). In such cases, habitat management could indirectly impact infection dynamics in wildlife and exposure risks for humans by influencing host community diversity and species abundance (Grosholz, 1993; Hite et al., 2016; Suzán et al., 2008).

Research investigating the impacts of habitat variation on the ecology of zoonotic pathogens has primarily focused on a small number of systems with well‐established human health implications (e.g., *Peromyscus*‐*Borrelia burgdorferi*; Adalsteinsson et al., 2018; Prusinski et al., 2006), but most zoonotic systems are still poorly understood. Drawing meaningful conclusions from pathogen data in wildlife, including rodents, can be difficult due to low prevalence that fluctuates over time and space, and many pathogens are limited to one or a few host species within a community (e.g., Cantoni et al., 2002; Essbauer et al., 2009; Salazar‐Bravo et al., 2004). As a result, broad inferences are often made based on model systems rather than specific host–pathogen ecology. For example, most information on American orthohantaviruses is inferred from studies on a select few common viruses despite 21 known orthohantaviruses occurring throughout North and South America and likely many more yet to be discovered (Mull et al., 2020, 2022).

In this study, we investigate how habitat management impacts wildlife and the viruses they carry. Rodent communities were compared among replicated grassland sites under different management regimes. We assessed the diversity and abundance of rodent species and how this translates to the presence and prevalence (through serology) of three groups of common rodent‐borne viruses: orthohantaviruses, arenaviruses, and orthopoxviruses (Forbes et al., 2014; Ogola et al., 2021). Since habitat management is usually designed to enhance species diversity, we hypothesize that rodent diversity and overall abundance will be higher in habitats with management reminiscent of natural ecosystems (i.e., prescribed burning). We hypothesize the opposite pattern for virus prevalence, with prevalence being lowest in burned habitats, as wildlife hosts of zoonotic pathogens tend to be more common in disturbed habitats (Keesing & Ostfeld, 2021).

## 2 MATERIALS AND METHODS

### 2.1 Study sites

**FIGURE 1:**
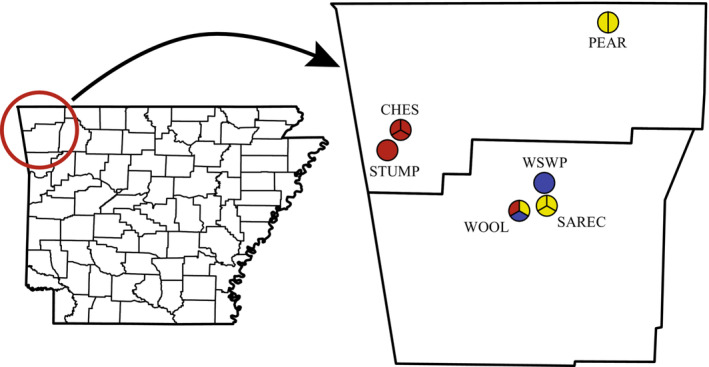
Map of grassland study sites in Benton and Washington Counties, Arkansas, USA. Wedges in circles represent individual sites at each grassland; red wedges indicate burned sites, yellow wedges indicate cut sites, and blue wedges indicate unmanaged sites. Each grassland site was given a short name for identification; CHES, Chesney Prairie Natural Area; STUMP, Stump's Prairie; PEAR, Pea Ridge National Military Park; SAREC, Milo J. Shult Agricultural Research & Extension Center; WOOL, Woolsey Wet Prairie; WSWP, Wilson Springs Wetland Preserve.

Rodents were captured in grasslands throughout Benton and Washington Counties, Arkansas, USA. This area lies near the edge of the historical tallgrass prairie ecoregion, and like other tallgrass prairie ecosystems, most of the landscape has been altered by humans, with few remnant prairies remaining. Instead, many of the modern grasslands in this region are restored prairies or nonprairie grasslands. Trapping was conducted at 13 sites within six distinct grasslands (Figure 1). Neighboring sites within the same grassland were considered separate areas as they are distinctly managed and separated by physical barriers (i.e., roadway, riparian habitat, and/or firebreak). Although these barriers do not act as complete physical barriers, they limit rodent movement among sites and distinguish separately managed parcels. Grasslands ranged in size from 6.7 to 32.6 ha, and distinct management sites within each grassland ranged in size from 1.5 to 23.6 ha. Site management was classified as one of three regimes: prescribed burning, reminiscent of natural ecosystem functioning (designated burn; five sites); haying, mowing, or other means of mechanical cutting, which result in managed yet artificial landscapes (designated cut; six sites); or no active management of vegetation, leading to heavy woody encroachment (designated unmanaged; two sites; Figure 1). Management regimes at the study sites have been continuous for several decades, and our results thus represent the long‐term effects of management regimes.

### 2.2 Rodent trapping and sampling

Rodent trapping was conducted once every 2 months at each site from June to November 2020 and April to July 2021. Because of the number of sites and distances between grasslands, sites were trapped in several groups over the course of the trapping month. To maintain consistency of time between trapping at each site, site groups were trapped in the same order each trapping month. For each trapping occasion, approximately 50 Sherman live traps (H. B. Sherman Traps, Inc.) were set for two consecutive nights approximately 10 m apart in a series of transect lines (see Table 1 for deviations of trap numbers). Traps were baited with a mixture of millet and black oil sunflower seeds and set at dusk. Traps were checked and captured rodents were processed the following morning. Initially, all rodents were euthanized for tissue collection except for species classified as species of conservation need by Arkansas Game and Fish Commission (*Reithrodontomys humulis*, *megalotis*, and *montanus*). Due to permit limitations, we were unable to euthanize all individuals of abundant species (*Reithrodontomoys fulvescens* and *Sigmodon hispidus*) in the fall 2020.

**TABLE 1:** Combined trapping effort and capture success among grasslands from 2020 to 2021.

Site	Management	Trap nights	Captures (%)	Seropositive (%)
CHES_A	Burn	458	60 (13.1)	8 (13.3)
CHES_B	Burn	458	70 (15.3)	2 (2.9)
CHES_C	Burn	438	66 (15.1)	10 (15.2)
STUMP	Burn	519	79 (15.22)	15 (19.0)
WOOL_A	Burn	500	73 (14.6)	1 (1.4)
PEAR_A	Cut	500	11 (2.2)	0
PEAR_B	Cut	500	7 (1.4)	0
SAREC_A	Cut	400	20 (5)	0
SAREC_B	Cut	400	24 (6)	0
SAREC_C	Cut	400	75 (18.8)	0
WOOL_B	Cut	460	17 (3.7)	2 (11.8)
WOOL_C	Unmanaged	400	14 (3.5)	0
WSWP	Unmanaged	520	100 (19.2)	0
Total		5953	616 (10.3)	38 (6.2)

*Note*: The number of captured and seropositive animals trapped for the duration of the study at each site, with percentages based on the number of trap nights for captures and the number of captures for seropositive columns.

Captured rodents were identified to species level based on morphology (pelage and lengths of the ear, tail, head/body, and hind foot; Sealander & Heidt, 1990; Reid, 2006). Visual inspection was used to determine sex and reproductive condition; males were considered to be reproductive if their testes were descended into the scrotum, and females were considered to be reproductive if their nipples were enlarged or lactating or if their vagina was perforate or plugged. Rodent blood samples were collected via either the submandibular vein directly into a microcentrifuge tube during processing and immediately placed on ice or a heart sample that was placed into phosphate‐buffered saline (PBS) during dissection (see below; Forbes et al., 2014). To promote efficiency and minimize handling time and associated distress to wild rodents, most rodent species were quickly euthanized via cervical dislocation without anesthetic; cotton rats were the exception due to their larger size and were anesthetized with inhalation isoflurane prior to cervical dislocation. Euthanized rodents were placed in individual labeled grip‐lock bags and stored in a cooler with ice while in the field. Rodents that were not euthanized were ear‐tagged and released at their point of capture following sample and data collection.

Euthanized rodents were stored in a −20°C freezer and later dissected under a biosafety hood. Tissue samples were collected aseptically using clean forceps and scissors and placed in sterilized microcentrifuge tubes. Hearts were placed in PBS solution to permit serology assays. All samples and specimens were stored at −20°C.

All animal handling and sampling procedures were approved by the University of Arkansas Institutional Animal Care and Use Committee protocol number 20028 and Arkansas Game and Fish Commission permit numbers 102820194 and 030820211. Additionally, sampling at Chesney Prairie Natural Area was also approved by Arkansas Natural Heritage Commission permit numbers S‐NHCC‐19‐025 and S‐NHCC‐21‐007.

### 2.3 Assays to detect antibodies against rodent viruses

Blood samples were tested for antibodies reactive to orthohantaviruses, arenaviruses, and orthopoxviruses using immunofluorescence assays, as previously described (Forbes et al., 2014; Kallio‐Kokko et al., 2006; Kinnunen et al., 2011). Briefly, samples were diluted in PBS and then incubated on slides with viral antigens followed by several wash cycles to remove unbound antibodies. Fluorescent polyclonal rabbit antimouse FITC conjugate was then added to the slides, which were again incubated and washed. Slides were examined under a fluorescence microscope for reactive antibodies. These serology assays are cross‐reactive within broad virus groups and therefore are effective and efficient approaches for nonspecific screening (e.g., Ogola et al., 2021).

### 2.4 Data analyses

All analyses were conducted in R 4.1.0 (R Core Team, 2021). We used a chi‐square test of independence to compare trapping success among management types. Renyi diversity profiles were used to compare several indices of rodent diversity among management regimes (Kindt, 2020; Tóthmérész, 1995). Additionally, an analysis of similarity (ANOSIM) test was used to determine if rodent community composition varied among management regimes using the Bray–Curtis dissimilarity index (Herlemann et al., 2016; Zorz, 2019).

Because rodents with antibodies against the focus virus groups were only detected in sites that were burned or cut, a chi‐square test of independence was used to test for differences in the total seroprevalence of all three viruses among habitat management regimes. Binomial generalized linear mixed models (GLMMs) with seropositivity as the response variable was then used to compare seroprevalence between burned and cut sites, with grassland and site identity as a nested random effect. Demographic data, including sex, reproductive status, abundance index (capture success), and their interactions were set as explanatory variables in the GLMMs. Because most seropositive cases were from rodents with antibodies against orthohantaviruses, we also used GLMMs to compare orthohantavirus seroprevalence alone among burned and cut sites. Two separate binomial GLMMs were used to analyze orthohantavirus seroprevalence from all sites within cotton rats and prairie voles (*M. ochrogaster*), as these two species accounted for the majority of seropositive rodents but have different life histories, including seasonal dynamics and mass (Brady & Slade, 2001). Large, reproductive male rodents are often disproportionately seropositive for orthohantaviruses (Douglass et al., 2007; Polop et al., 2010), so explanatory variables for species‐level GLMMs included mass, sex, reproductive status, abundance index, and their interactions. Finally, a Poisson GLMM, again using seropositivity as the response variable, was used to compare seroprevalence by trap success at each site and trapping occasion, with trapping occasion as a random effect and grassland and site identity as a nested random effect. GLMMs were conducted using the *lme4* package (Bates et al., 2022); all other statistical analyses were conducted using base R (R Core Team, 2021).

It is worth noting that although sites were grouped according to management regime, some differences in management schedules, site history, and biogeochemical factors were unavoidable and created heterogeneity within group categories. In particular, three of the five burned sites were burned every 3 years and the other two were burned annually. These differences were unavoidable due to the study design, akin to a natural experiment. However, potential differences due to site heterogeneity within management categories were assessed to validate groupings; no differences in rodent abundance, rodent diversity, or seroprevalence were detected between burn frequencies (Appendix 1). Despite several replicates of burned and cut sites, only two unmanaged sites were available in this study, as these habitats change drastically with the onset of management and are prone to ecological succession in the prolonged absence of management.

## 3 RESULTS

**FIGURE 2:**
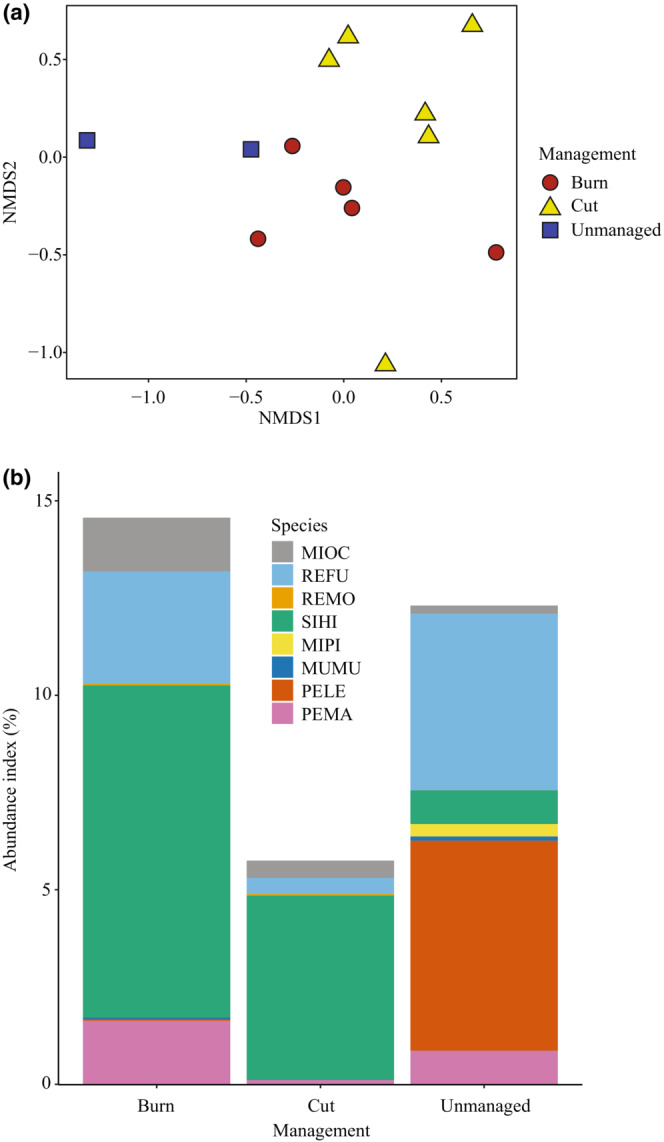
Comparison of community composition using (a) nonmetric multidimensional scaling and (b) an abundance index of each species per management regime. The abundance index is calculated as % capture rate for the duration of the study. Species identifiers are MIOC, *Microtus ochrogaster*; MIPI, *Mi. pinetorum*; MUMU, *Mus musculus*; PELE, *Peromyscus leucopus*; PEMA, *P. maniculatus*; REFU, *Reithrodontomys fulvescens*; REMO, *R. montanus*; SIHI, *Sigmodon hispidus*.

A total of 616 rodents were captured across 5953 trap nights (Table 1), and no tagged animals were recaptured. Capture success ranged from 0% to 45% depending on site and season. We captured eight different rodent species throughout the study, with 2–6 different species at individual sites. Rodent community composition varied moderately but significantly among management regimes (ANOSIM *p* < .03, *R* = .54; Figure 2a).

**FIGURE 3:**
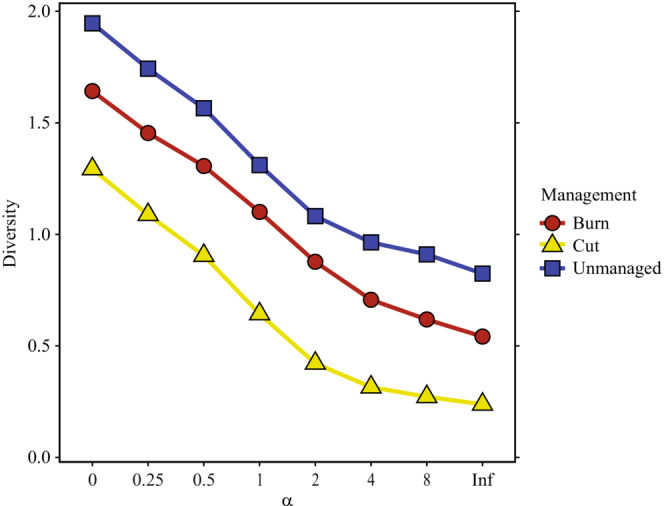
Renyi diversity profiles among grasslands with burned, cut, or unmanaged management regimes. Lower alpha values are heavily weighted by evenness, and higher alpha values are heavily weighted by the abundance of dominant species.

Capture success varied among management regimes (χ^2^ = 91.07, *p* < .001; Table 1). Success was higher at burned and unmanaged sites than at cut sites (both *p* < .001; Figure 2b) and did not differ between burned and unmanaged sites (*p* = .16). Rodent diversity also varied among habitat management regimes, with unmanaged and burned sites having higher rodent diversity than cut sites across all Renyi alpha values (Figure 3). Rodent diversity was similar between burned and unmanaged sites, though unmanaged sites consistently had higher rodent diversity (Figure 3).

A total of 38 rodents (6.2%) were seropositive for one of the tested virus groups (34 orthohantavirus, three arenavirus, one orthopoxvirus; Table 2). All seropositive animals were caught at burned sites except one orthohantavirus‐seropositive fulvous harvest mouse and one orthohantavirus‐seropositive cotton rat (Table 1). The majority of seropositive individuals were cotton rats and prairie voles with orthohantavirus antibodies (Table 2).

**TABLE 2:** Total number of each species captured among all sites that were seropositive for orthohantavirus, arenavirus, and orthopoxvirus.

Scientific name	Common name	Captures	Seropositive (%)		
			Orthohantavirus	Arenavirus	Orthopoxvirus
*Microtus ochrogaster*	Prairie vole	47	7 (14.9)	0	0
*Microtus pinetorum*	Woodland vole	3	0	0	0
*Mus musculus*	House mouse	2	0	1 (50)	0
*Peromyscus leucopus*	White‐footed mouse	51	0	0	0
*Peromyscus maniculatus*	Deer mouse	50	0	0	0
*Reithrodontomys fulvescens*	Fulvous harvest mouse	122	1 (0.8)	2 (1.6)	0
*Reithrodontomys montanus*	Plains harvest mouse	2	0	0	0
*Sigmodon hispidus*	Hispid cotton rat	339	26 (7.7)	0	1 (0.3)
Total		616	34 (5.5)	3 (0.5)	1 (0.2)

**FIGURE 4:**
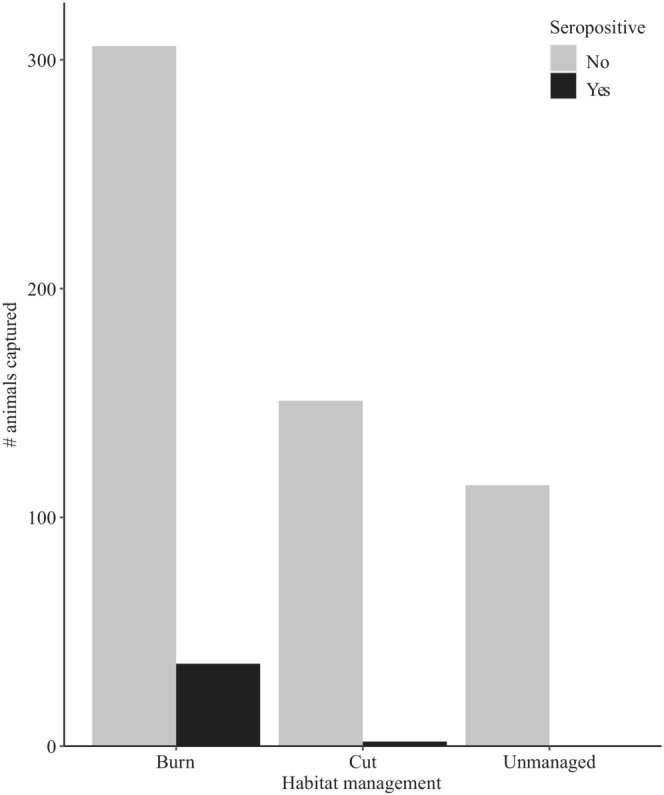
Number of rodents caught among sites with burned, cut, or unmanaged management regimes for the duration of the study that were seropositive or seronegative for any tested virus group (orthohantaviruses, arenaviruses, or orthopoxviruses).

Complete processing data were collected for 609/616 rodents captured for infection analyses. Based on the Chi‐square test for independence, virus seroprevalence varied among management types (χ^2^ = 24.69, *p* < .001; Figure 4), with a higher proportion of seropositive rodents in burned sites than cut or unmanaged sites (both *p* < .001). No difference in seroprevalence was detected between cut and unmanaged sites (*p* = .61). The most parsimonious GLMM comparison between burned and cut sites only included the type of habitat management and reproductive condition (Table A1 in Appendix 1). This model confirmed that seropositive rodents were more common in burned sites than cut sites (*p* < .04) and that reproductive individuals were more likely to be seropositive than non‐reproductive individuals (*p* < .01). Unsurprisingly, orthohantavirus seroprevalence was similar to overall seroprevalence, with burned sites and reproductive condition being predictors of orthohantavirus seropositivity (*p* < .05 and *p* < .01, respectively). Additionally, higher rodent abundance was associated with higher seroprevalence at a given site and trapping occasion (*p* < .03).

**FIGURE 5:**
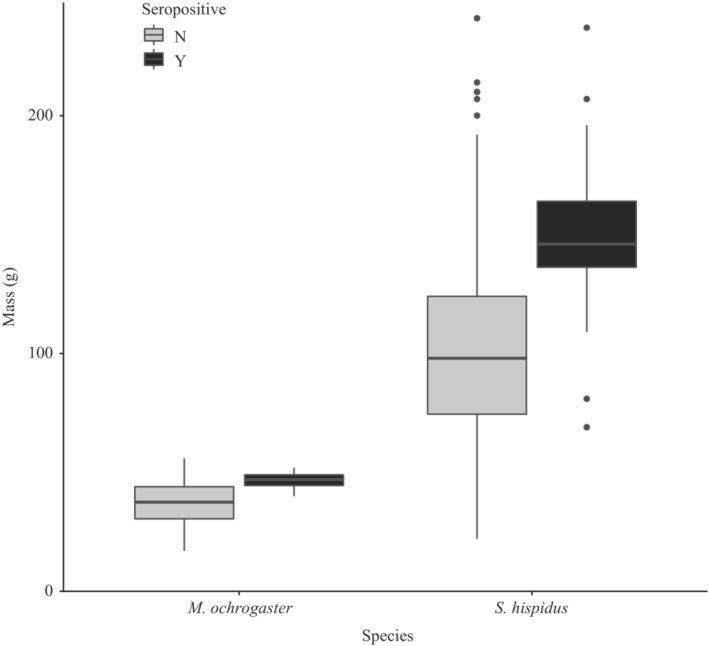
Mass of *Microtus ochrogaster* (prairie voles) and *Sigmodon hispidus* (hispid cotton rats) from all sites that were seropositive or seronegative for orthohantaviruses.

Similar to the GLMM with all individuals, sex and abundance were not important predictors in seroprevalence for cotton rats or prairie voles individually (Tables A2 and A3 in Appendix 1). However, the reproductive condition was not a variable in the most parsimonious models at the species level. Heavier individuals of both cotton rats and prairie voles were more likely to be seropositive than lighter individuals (*p* < .001 and *p* < .04, respectively; Figure 5).

## 4 DISCUSSION

We demonstrate that habitat management regimes lead to differences in rodent community assemblages, species abundances, and subsequently, viral infection dynamics. Burned habitats produced the highest overall quality of grassland rodent communities, with high rodent diversity, overall abundance, and a relatively high proportion of grassland species. In comparison, cut habitats had low diversity and abundance but a high proportion of grassland species, and unmanaged habitats had high diversity and abundance but a low proportion of grassland species. Most of the virus seropositive rodents in this study were grassland species found in burned sites. Our findings highlight the advantages and disadvantages of different grassland habitat management regimes for biodiversity indicators and the importance of identifying these trade‐offs.

We found that burned and unmanaged sites had similar rodent diversity and overall abundance but differed in the relative proportions of grassland species. The high proportion of generalist and forest‐specialist species, particularly *Peromyscus leucopus*, in unmanaged sites is indicative of the consequences of habitat degradation in the absence of grassland management, particularly from woody encroachment and loss of non‐woody diversity (Brunsell et al., 2017; Miller et al., 2000). Although some studies have shown that burning increases the relative abundance of habitat generalists (e.g., Manyonyi et al., 2020; Zúñiga et al., 2020), such outcomes generally represent the immediate effects of fire, as opposed to prolonged effects from a decade or more of management. Indeed, rodent diversity at several of our burned sites varied considerably from a previous assessment shortly after active management began (Nelson, 2005), most notably by an increase in our study of prairie voles and fulvous harvest mice, two grassland species (Table 2). Similar positive long‐term effects of prescribed burning on habitat availability and species richness in grasslands have been identified for other wildlife taxa, including insects and elk (Bargmann et al., 2015; Podgaiski et al., 2017; Van Dyke & Darragh, 2006).

Cut sites, on the other hand, had lower diversity and abundance but a higher relative proportion of grassland species than burned and unmanaged sites. Hayed or mowed fields generally have low vegetation diversity compared with other grasslands (Faria et al., 2018), which limits ecosystem functioning across a variety of wildlife (Wan et al., 2020). Species capable of using the dominant vegetation in these areas have access to abundant resources and their population sizes can become very large. For example, one of our cut study sites had the second‐highest abundance, and 87% of the rodents captured throughout the study were hispid cotton rats (Table 1). However, abundance in cut sites varied considerably depending on whether the field was recently cut or not, reducing the stability of wildlife populations in these areas. Grazing by livestock is another method to manage habitats that are often considered analogous to cutting vegetation. Although grazing is a more natural means of removing vegetation and can produce more diverse vegetation communities than haying or mowing (Tälle et al., 2016), intensive grazing drastically reduces rodent abundance (La Morgia et al., 2015; Yarnell et al., 2007). Thus, while light grazing may increase wildlife diversity, intensive grazing is likely similar to, or worse than, cutting for rodent diversity.

Differences in seroprevalence among management regimes are likely driven by differences in rodent abundance, particularly of grassland species, at these sites. All seropositive animals in this study were grassland species, and orthohantaviruses are characterized by density‐dependent transmission and high host specificity (McGuire et al., 2016; Mills et al., 1999). In particular, cotton rats, the reservoir host of Black Creek Canal virus (orthohantavirus; Rollin et al., 1995), were the most commonly trapped species and accounted for the majority of seropositive individuals for all viruses and orthohantaviruses specifically (Table 2 and Table A4 in Appendix 1). Although fire refugia may have impacted virus transmission (Albery et al., 2021), high virus prevalence in dominant rodent species in natural, pristine habitats is consistent with recent studies from South America (Burgos et al., 2021; Tirera et al., 2021). Interestingly, we identified no seropositive deer mice or white‐footed mice, the reservoir hosts of Sin Nombre virus and New York virus (both orthohantaviruses that would be detected with our serology assay; Yamada et al., 1995), respectively (Childs et al., 1994; Hjelle et al., 1995), despite relatively high orthohantavirus seroprevalence in the similarly‐abundant prairie voles (Table 2). Burned sites were therefore the only habitat capable of supporting the orthohantavirus host populations in our study area.

In addition to the management regime, several other variables influenced infection dynamics in this study. Although rodent abundance was important in predicting seroprevalence at each site for the duration of the study, it was not useful in predicting seroprevalence at sites on individual trapping occasions. This is likely due to a time lag effect, where prevalence is impacted earlier rather than current density (Adler et al., 2008; Yates et al., 2002). Heavier individuals were more likely to be seropositive for orthohantavirus (Figure 5), consistent with other studies that show positive relationships between mass and orthohantavirus seroprevalence (Glass et al., 1998; Walsh et al., 2007). There were too few rodents seropositive for arenaviruses or orthopoxviruses for statistical evaluation, but the high demographic variety of arenavirus hosts in this study (Table A4 in Appendix 1) corroborates our understanding of American arenavirus host demography, which includes individuals of both sexes and all age classes (Milazzo et al., 2008, 2013). Conversely, the low seroprevalence of orthopoxviruses was surprising, as these viruses are commonly found in diverse wild rodent species from other geographical areas (e.g., Forbes et al., 2014; Kinnunen et al., 2011; Ogola et al., 2021), though relatively little is known about orthopoxviruses in American rodents (but see Emerson et al., 2009).

Overall, our study evaluates the impacts of long‐term habitat management on wildlife and their pathogens. High‐intensity grassland management (i.e., prescribed burning) generated high diversity and abundance of rodents. Burned sites also had the highest virus seroprevalence and were the only sites where rodents with antibodies against two of the three virus groups were detected. Biodiversity is crucial for healthy ecosystem functioning, and these results provide empirical evidence that can inform grassland restoration and ongoing management strategies, especially in prairie ecoregions.

## AUTHOR CONTRIBUTIONS

**Nathaniel Mull:** Conceptualization (lead); data curation (lead); formal analysis (lead); investigation (lead); methodology (equal); supervision (supporting); visualization (lead); writing – original draft (lead); writing – review and editing (equal). **Amy Schexnayder:** Investigation (equal); writing – original draft (equal); writing – review and editing (supporting). **Abigail Stolt:** Investigation (equal); writing – original draft (equal); writing – review and editing (supporting). **Tarja Sironen:** Investigation (supporting); methodology (equal); resources (equal); supervision (supporting); writing – original draft (supporting); writing – review and editing (supporting). **Kristian M. Forbes:** Funding acquisition (lead); methodology (equal); resources (equal); supervision (lead); writing – original draft (supporting); writing – review and editing (equal).

## ACKNOWLEDGEMENTS

We thank Reilly Jackson, Ellery Lassiter, Shannon Kitchen, Aaron Norris, C. Houston Lamb, and Alexia Hernandez for assistance with rodent trapping. We also thank Sanna Mäki and other staff of the Viral Zoonosis Research Unit from the University of Helsinki for assistance with serology tests. We are very grateful to the grassland land managers for allowing us to collect samples from and for managing the sites used in this study, including Arkansas Natural Heritage Commission, City of Fayetteville, Joe Woolbright, Milo J. Shult Agricultural Research & Extension Center, Northwest Arkansas Land Trust, and Pea Ridge National Military Park.

## FUNDING INFORMATION

This research was partly supported by NSF DEB 1911925.

## CONFLICT OF INTEREST STATEMENT

The authors declare that they have no conflict of interest.

## Data Availability

The data that support the findings of this study are openly available in Dryad at https://doi.org/10.5061/dryad.6t1g1jx29.

## APPENDIX 1

### 1.1

As a form of validation, we compared capture success, rodent diversity, and rodent seroprevalence between sites that were burned every 3 years and sites that were burned annually to verify that burn frequency did not influence our results. Capture success was compared using a Chi‐square test for independence (χ^2^ = 0.063, *p* = .80). Rodent diversity was compared using a linear mixed effects model (*lme4* package in R) with each site's Shannon index as the response variable, burn frequency as the explanatory variable, and prairie as a random effect (*p* = .60). Rodent seroprevalence was compared using a binomial GLMM (*lme4* package in R) with individual seroprevalence as the explanatory variable, burn frequency as the explanatory variable, and prairie as a random effect (*p* = .89; Tables A1, A2, A3, and A4).

**TABLE A1:** AIC values for GLMMs comparing seroprevalence of all rodents between burned and cut sites.

Variables	AIC
Management + Reproductive	239.8
Management * Reproductive	241.2
Management + Sex + Reproductive	241.4
Management * Success + Reproductive	242.7
Management * Reproductive + Success	242.8
Management + Sex * Reproductive	242.8
Management * Reproductive + Sex	242.9
Management + Success + Sex + Reproductive	243.0
Management * Sex + Reproductive	243.2
Management * Success + Sex + Reproductive	244.4
Management * Reproductive + Success + Sex	244.4
Management * Sex + Success + Reproductive	244.8
Management + Success	249.0
Management	249.1
Management + Success + Sex	250.2
Management + Sex	250.5
Management * Success	250.7
Management * Success + Sex	251.9
Management * Sex + Success	251.9
Management * Sex	252.2

**TABLE A2:** AIC values for GLMMs comparing orthohantavirus seroprevalence of hispid cotton rats (*Sigmodon hispidus*).

Variables	AIC
Mass	133.3
Mass + Abundance	133.7
Mass * Abundance	134.2
Mass * Reproductive	135.0
Mass + Reproductive	135.0
Mass + Sex + Abundance	135.5
Mass + Reproductive + Abundance	135.6
Mass * Reproductive + Abundance	135.6
Mass * Abundance + Sex	136.0
Mass * Abundance + Reproductive	136.1
Mass * Reproductive + Sex	136.9
Mass + Sex + Reproductive	137.0
Mass + Sex * Reproductive	137.3
Mass + Reproductive * Sex	137.3
Mass * Sex + Abundance	137.4
Mass * Reproductive + Sex + Abundance	137.4
Mass + Sex * Abundance	137.5
Mass + Sex + Reproductive + Abundance	137.5
Mass + Sex * Reproductive + Abundance	137.8
Mass * Abundance + Sex + Reproductive	138.0
Mass + Sex + Reproductive * Abundance	138.1
Mass * Sex + Reproductive	138.9
Mass * Sex + Reproductive + Abundance	139.4
Mass * Sex * Reproductive	140.3
Mass * Abundance * Reproductive + Sex	140.7
Mass * Sex * Reproductive + Abundance	140.7
Mass * Sex * Abundance + Reproductive	143.8
Mass * Sex * Reproductive * Abundance	147.2

**TABLE A3:** AIC values for GLMMs comparing seroprevalence of prairie voles (*Microtus ochrogaster*).

Variables	AIC
Mass * Sex	31.3
Mass	31.3
Mass + Reproductive	31.5
Mass + Abundance	31.8
Mass + Success + Reproductive	32.5
Mass + Sex	32.5
Mass + Sex + Abundance	32.7
Mass * Sex + Reproductive	32.9
Mass * Abundance + Reproductive	33.2
Mass + Sex + Reproductive	33.2
Mass * Abundance	33.2
Mass * Abundance + Sex	33.7
Mass + Sex + Abundance + Reproductive	33.8
Mass + Sex * Abundance	34.0
Mass * Sex + Abundance + Reproductive	34.1
Mass * Abundance + Sex + Reproductive	34.5
Mass + Sex * Abundance + Reproductive	35.4

*Note*: Interaction effects between reproductive and other variables could not be computed because all seropositive prairie voles were in reproductive condition.

**TABLE A4:** Individual demographic information for seropositive rodents.

Virus	Species	Sex	Reproductive	Mass (g)	Site
H	*Microtus ochrogaster*	F	Yes	47	CHES_C
H	*Microtus ochrogaster*	F	Yes	48	CHES_C
H	*Microtus ochrogaster*	F	Yes	52	CHES_A
H	*Microtus ochrogaster*	M	Yes	40	CHES_A
H	*Microtus ochrogaster*	M	Yes	44	CHES_C
H	*Microtus ochrogaster*	M	Yes	45	CHES_B
H	*Microtus ochrogaster*	M	Yes	50	CHES_A
H	*Reithrodontomys fulvescens*	M	Yes	11	WOOL_A
H	*Sigmodon hispidus*	F	No	126	STUMP
H	*Sigmodon hispidus*	F	No	133	CHES_C
H	*Sigmodon hispidus*	F	No	136	STUMP
H	*Sigmodon hispidus*	F	Yes	81	WOOL_B
H	*Sigmodon hispidus*	F	Yes	155	STUMP
H	*Sigmodon hispidus*	F	Yes	167	CHES_C
H	*Sigmodon hispidus*	F	Yes	169	CHES_A
H	*Sigmodon hispidus*	F	Yes	174	STUMP
H	*Sigmodon hispidus*	M	No	139	STUMP
H	*Sigmodon hispidus*	M	No	153	CHES_C
H	*Sigmodon hispidus*	M	No	207	CHES_C
H	*Sigmodon hispidus*	M	Yes	69	CHES_C
H	*Sigmodon hispidus*	M	Yes	109	STUMP
H	*Sigmodon hispidus*	M	Yes	129	CHES_C
H	*Sigmodon hispidus*	M	Yes	137	STUMP
H	*Sigmodon hispidus*	M	Yes	139	CHES_A
H	*Sigmodon hispidus*	M	Yes	140	STUMP
H	*Sigmodon hispidus*	M	Yes	142	CHES_A
H	*Sigmodon hispidus*	M	Yes	146	WOOL_B
H	*Sigmodon hispidus*	M	Yes	146	STUMP
H	*Sigmodon hispidus*	M	Yes	148	CHES_B
H	*Sigmodon hispidus*	M	Yes	159	STUMP
H	*Sigmodon hispidus*	M	Yes	164	STUMP
H	*Sigmodon hispidus*	M	Yes	164	STUMP
H	*Sigmodon hispidus*	M	Yes	196	STUMP
H	*Sigmodon hispidus*	M	Yes	237	STUMP
A	*Mus musculus*	M	Yes	19	CHES_A
A	*Reithrodontomys fulvescens*	F	Yes	16	CHES_V
A	*Reithrodontomys fulvescens*	M	Yes	9	STUMP
P	*Sigmodon hispidus*	M	Yes	133	CHES_A

*Note*: For virus, H = orthohantavirus, A = arenavirus, and P = orthopoxvirus.
